# Noble Metals for Modern Implant Materials: MOCVD of Film Structures and Cytotoxical, Antibacterial, and Histological Studies

**DOI:** 10.3390/biomedicines9080851

**Published:** 2021-07-21

**Authors:** Svetlana I. Dorovskikh, Evgeniia S. Vikulova, Elena V. Chepeleva, Maria B. Vasilieva, Dmitriy A. Nasimov, Eugene A. Maksimovskii, Alphiya R. Tsygankova, Tamara V. Basova, David S. Sergeevichev, Natalya B. Morozova

**Affiliations:** 1 Nikolaev Institute of Inorganic Chemistry, Siberian Branch, Russian Academy of Sciences, 3 Acad. Lavrentiev Ave., 630090 Novosibirsk, Russia; reter16@yandex.ru (S.I.D.); lazorevka@mail.ru (E.S.V.); eugene@niic.nsc.ru (E.A.M.); alphiya@niic.nsc.ru (A.R.T.); basova@niic.nsc.ru (T.V.B.); 2 E. Meshalkin National Medical Research Center of the Ministry of Health of the Russian Federation, 15 Rechkunovskaya Str., 630055 Novosibirsk, Russia; e_chepeleva@meshalkin.ru (E.V.C.); vasilievam@yandex.ru (M.B.V.); d_sergeevichev@meshalkin.ru (D.S.S.); 3 Rzhanov Institute of Semiconductor Physics, Siberian Branch, Russian Academy of Sciences, 15 Acad. Lavrentiev Ave., 630090 Novosibirsk, Russia; nasimov@isp.nsc.ru

**Keywords:** titanium-alloy implants, iridium, platinum, gold, silver, chemical vapor deposition, thin films and nanoparticles, cytological, biochemical and cytokine blood composition, histological study

## Abstract

This work is aimed at developing the modification of the surface of medical implants with film materials based on noble metals in order to improve their biological characteristics. Gas-phase transportation methods were proposed to obtain such materials. To determine the effect of the material of the bottom layer of heterometallic structures, Ir, Pt, and PtIr coatings with a thickness of 1.4–1.5 μm were deposited by metal–organic chemical vapor deposition (MOCVD) on Ti_6_Al_4_V alloy discs. Two types of antibacterial components, namely, gold nanoparticles (AuNPs) and discontinuous Ag coatings, were deposited on the surface of these coatings. AuNPs (11–14 nm) were deposited by a pulsed MOCVD method, while Ag films (35–40 nm in thickness) were obtained by physical vapor deposition (PVD). The cytotoxic (24 h and 48 h, toward peripheral blood mononuclear cells (PBMCs)) and antibacterial (24 h) properties of monophase (Ag, Ir, Pt, and PtIr) and heterophase (Ag/Pt, Ag/Ir, Ag/PtIr, Au/Pt, Au/Ir, and Au/PtIr) film materials deposited on Ti-alloy samples were studied in vitro and compared with those of uncoated Ti-alloy samples. Studies of the cytokine production by PBMCs in response to incubation of the samples for 24 and 48 h and histological studies at 1 and 3 months after subcutaneous implantation in rats were also performed. Despite the comparable thickness of the fibrous capsule after 3 months, a faster completion of the active phase of encapsulation was observed for the coated implants compared to the Ti alloy analogs. For the Ag-containing samples, growth inhibition of *S. epidermidis, S. aureus, Str. pyogenes, P. aeruginosa*, and *Ent. faecium* was observed.

## 1. Introduction

The modern implant intended for use in orthopedic, reconstructive, and oncological practices is a complex structure that performs various functions in the body. The main properties that any of the implant materials should possess, first of all, reliability and safety of their use, including biocompatibility, osseointegration, and antibacterial properties [1]. Implants must be inert to living tissues, be not carcinogenic, have a sufficient margin of mechanical strength, and be resistant to the internal environment of the body [2,3,4]. Biocompatibility and prolonged antibacterial effect are of particular importance, where patients have reduced immunity and a tendency to infectious complications. Additionally, implants must perform their functions without replacement for a long time [5,6,7,8,9]. An important point is the chemical composition of the surface and the relief of modern biomaterials that determine the implant properties listed above.

Traditional materials for the manufacture of implants remain metals characterized by biological compatibility, in particular, special grades of stainless steel, as well as titanium and its alloys [9,10,11,12,13,14]. However, it should be noted that any implant has certain disadvantages. Despite the high corrosion resistance of metal implants or the formation of a protective oxide film in the case of titanium-containing alloys, the main problem of their use is the gradual leaching of metal ions, which leads to the development of toxic or allergic reactions, as well as metallosis phenomena [15,16,17]. Other serious complications of reconstructive surgery are infectious and inflammatory. Such complications often occur due to bacterial contamination of the implant surface during surgical implantation. Bacteria can adhere to various biomaterials and colonize them. Uncontrolled bacterial growth can lead to the formation of bacterial biofilms, which protect the microorganisms from the host’s defense mechanisms and make antibiotic treatment difficult or even completely ineffective [18]. Usually, an infected implant can lead to serious complications, including surgical revision, surgical treatment of the wound, and long-term antibacterial therapy [19]. In this regard, a necessary step in improving the effectiveness of permanent metal implants is the development of coatings that can improve the biointegrating properties of implants and provide a prolonged antibacterial effect. One approach is to use silver coatings and nanoparticles, antibiotics, or bioactive ceramics. However, the risk of toxic reactions such as argyrosis (i.e., a condition caused by prolonged deposition of silver in the body) and the limited duration of action (in the case of antibiotics) do not allow us to talk about a complete solution of this problem [20,21,22,23,24].

Recently, researchers have begun to use a new approach, which consists of modifying the surface of implant materials with thin films of platinum group metals. Such metals have exceptional chemical inertia, high biological compatibility, and corrosion resistance. To enhance bactericidal properties, gold and silver nanoparticles are deposited onto the surface of metal implants. Recent results [15] obtained for silver, gold, and platinum coatings demonstrate the significant potential of these materials. Authors of these works studied the mechanism of silver ion release by using the principle of a sacrificial anode for thin-film systems consisting of microstructured Ag films on coatings with a “more noble” base layer (gold, Ir, Pt, or Pd). Ag on noble metals has been shown to corrode much faster than Ag on Ti. It creates increased antimicrobial activity compared to the system consisting of Ag and “the less noble” metal Ti, which increases the osseointegrative and anti-inflammatory effect of the studied systems. Thus, the use of coatings based on platinum metals with gold and silver nanoparticles may increase the biocompatibility of metal implants and make it possible to control the antibacterial activity. The most promising combination of metals for this “multifunctional” material is a mixture of gold, silver, platinum, and iridium, and the first stage of research is the study of the biocompatibility of these film heterostructures.

Evaluation of the biomedical properties of film mono- and heterostructures includes a number of studies, the key of which are in vitro laboratory tests for cytotoxicity and antibacterial activity. The cytotoxicity of film structures is the biological property of materials to cause the death of eukaryotic cells upon contact through a biological fluid. This property characterizes the bioinertness of the sample surface; the more viable cells remain after cytotoxic tests with experimental implant coatings, the higher the probability of successful biointegration of the sample will be after implantation in vivo.

An additional and important test is the study of the secretion of cytokines and chemokines by peripheral blood cells when interacting with modified surfaces. Such studies allow for a more detailed assessment of pro- and anti-inflammatory leukocyte reactions. The study of the antibacterial activity of samples is a type of cytotoxicity studies, but it is advisable to talk about it only in relation to pathogenic bacteria. The antibacterial activity of the surface determines the presence of anti-inflammatory properties of the implant material. Performing these tests will allow us to choose the most promising coatings with minimal cytotoxicity and good antibacterial activity from several variants of surfaces with different types of film structures.

In this work, the processes of obtaining monometallic and composite iridium- and platinum-containing thin films, as well as their composites with gold nanoparticles and silver coatings, by metal–organic chemical vapor deposition (MOCVD) on Si(100) and Ti-alloy substrates are developed. This precision method was chosen due to the ability to deposit noble-metal coatings with a given composition, microstructure, and surface relief at relatively low temperatures.

Metal–organic complexes of platinum, iridium, and gold with beta-diketonate and cyclooctadiene ligands were used as precursors, for which synthetic approaches were developed and the thermal properties were studied in detail [25]. During the deposition process, metal coatings were formed as a result of a thermally activated chemical reaction of precursor vapors with a reactant gas on the substrate surface, with further removal of gaseous byproducts from the reaction zone. Silver coatings were prepared by physical vapor deposition (PVD).

The deposited film samples were examined in detail in order to determine their following properties and characteristics:

- composition, microstructure, and surface characteristics;
- cytotoxic properties in the culture of donor peripherical blood mononuclear cells using an XTT test;
- cytokine composition of the culture medium during cocultivation of mononuclear blood cells with the studied samples;
- antibacterial properties in bacterial cultures *S. epidermidis, S. aureus, Str. pyogenes, P. aeruginosa*, and *Ent. faecium* via a disc diffusion method;
- evaluation of in vivo biocompatibility and histological examinations.

## 2. Materials and Methods

### 2.1. Deposition of Ir, Pt, and IrPt Metal Coatings and Au and Ag Nanoparticles

Volatile complexes of iridium, platinum, and gold with pentane-1,3-dionate (acac), 2,2,6,6-trimethylheptane-3,5-dionate (thd), and cyclooctadiene-1,3 (cod) ligands, [Ir(cod)(acac)], [Pt(acac)_2_], and [(CH_3_)_2_Au(thd)], were synthesized and characterized in detail in [26,27,28,29,30] for the deposition of metal coatings and nanoparticles. The details of synthesis and characterization of these precursors are presented in the Supplementary Materials. MOCVD experiments were carried out in a vertical reactor with cold walls [31] at reduced pressure (1.7–2.1 Torr). Single-crystal Si(100) plates 10 × 10 mm^2^ (hereafter Si, Alga-SW, Novosibirsk, Russia) and discs made of medical alloy Ti_6_Al_4_V (hereafter Ti-alloy, thickness 2 mm, diameter 10 mm, Baoji Chenyuan Metal Materials Co., Ltd., Baoji, China) were used as substrates. Thus, the following metal coatings were deposited: Ir, Pt, and IrPt on Si (hereafter Pt(Si), Ir(Si), and PtIr(Si)) and Ti-alloy substrates (hereafter Pt, Ir, and PtIr).

Ir coatings were obtained at a deposition temperature of 310 °C, evaporator temperature of 110 °C (partial pressure of [Ir(cod)(acac)] was 0.02 Torr according to the equation ln(p, atm) = 23.4 − 14,136/T(K) [25]), and flow rates of 2 L/h for both carrier gas (argon) and reactant gas (oxygen). Pt coatings were obtained at a deposition temperature of 310 °C, evaporator temperature of 150 °C (partial pressure of [Pt(acac)_2_] was 0.03 Torr according to the equation ln(p, atm) = 23.03 − 12,817/T(K) [25]), and argon and oxygen flow rates of 2 L/h and 1 L/h, respectively. PtIr coatings were obtained at a deposition temperature of 310 °C and argon and oxygen flow rates of 1.5 L/h and 1 L/h. To obtain a stoichiometric metal ratio (1:1) in the coatings, the evaporation temperatures were set to provide the same precursor partial vapor pressure of 0.11 Torr, specifically, 165 °C for [Pt(acac)_2_] and 131 °C for [Ir(cod)(acac)] [25].

Au nanoparticles were deposited by pulsed metal–organic chemical vapor deposition (pulsed-MOCVD) on Si, Ti alloy, and Pt-, Ir-, and PtIr-coated Ti-alloy substrates (hereafter Au(Si), Au, Au/Pt, Au/Ir, and Au/PtIr, respectively) from [(CH_3_)_2_Au(thd)] in the reactor described in our previous work [32]. The experimental parameters were fixed as follows: the deposition temperature was 250 °C; the evaporator temperature was 70 °C (the saturated vapor pressure is given by the equation ln(p, atm) = 36.61 − 15,231/T(K) [33]); the ratio of reactant gaseous mixture (Ar/H_2_) was 2; the total reactor pressure was ~8 Torr, the number of cycles was 10. Each cycle involved the following steps: pump of the reactor, input of the precursor vapor, and input of the carrier and reaction gases, decomposition reaction, and pump of the reactor. The time of one cycle was 2 min.

Ag nanoparticles were deposited by thermal evaporation of metallic Ag (2 mg) in vacuum (*p* = 10^−6^ Torr) using a VUP-5 PVD installation on Si and Ti substrates and Pt-, Ir-, PtIr-coated Ti-alloy substrates (hereafter Ag(Si), Ag, Ag/Pt, Ag/Ir, and Ag/PtIr, respectively). The evaporator temperature was 962 °C (silver melting point), and the substrate temperature was 50 °C. The deposition procedures were carried out until the Ag was completely evaporated.

### 2.2. X-ray Diffraction Analysis

The phase composition of the coatings was determined by X-ray diffraction (XRD) using a Shimadzu XRD-7000 diffractometer, Japan. The XRD patterns were recorded with a step of 2θ = 0.0143° and the accumulation time of 5 s with CuKα radiation (Ni filter, linear detector OneSight, range of 2θ = 10–65°) or CoKα radiation (Fe filter, linear detector OneSight, range of 2θ = 10–80°), using the software PowderCell 2.4 and WINFIT 1.2.1. The results of all measurements were transferred to CuKα radiation for easy comparison. The diffraction patterns were indexed according to the Powder Diffraction File [34]. The calculation of the unit cell parameter (UCP) of Ir was performed in the PowderCell 2.4 program [35] taking into account the internal (Si substrate) or external (polycrystalline silicon) standards. The UCPs of Au were calculated from the position of the (111) peak. The coherent scattering regions (CSR) were calculated using the Scherrer equation, taking into account the half-widths of the polycrystalline silicon standard.

### 2.3. Scanning Electron Microscopy

Microstructural features of the surface and cleavage of the deposited coatings were studied using scanning electron microscopy (SEM, JEOL-ISM 6700 F microscope, Tokyo, Japan) and HITACHI UHR FE-SEM SU8200.

### 2.4. X-ray Photoelectron Spectroscopy

Quantitative data on the coating composition were obtained using X-ray photoelectron spectroscopy (XPS, SPECS spectrometer, Germany, PHOIBOS-150-MCD-9 analyzer, FOCUS-500 monochromator, Al Kα radiation, hv = 1486.74 eV, 200 W). The binding energy scale (E_b_) was calibrated using the positions of the peak energy levels of the Au 4f_7/2_ (E_b_ = 84.0 eV) and Cu 2p_3/2_ (E_b_ = 932.67 eV). To remove the surface layer, the samples were bombarded with Ar^+^ ions with energy of 2.5 keV for 10 min (5 nm depth). The spectra were processed in the CASA program (Japan) using the Voigt function. The background was taken into account using the Shirley method. Parameters proposed by Pfeifer et al. [36] were used for the fitting of Ir 4f peaks.

### 2.5. Atomic Emission Spectroscopy

The content of gold and silver in the investigated samples was determined by inductively coupled plasma atomic emission spectroscopy (ICP-AES) using a high-resolution spectrometer iCAP 6500 (Thermo Fisher Scientific, Waltham, MA, USA). The sample solution was injected into the plasma through a nebulizer of SeaSpray type using a peristaltic pump with the rate of 0.7 mL/min. The registration was performed at the axial observation of plasma under the conditions recommended by the spectrometer manufacturer: cooling argon flow—12 L/min, secondary—0.5 L/min, registration time—5 s, and power supplied to ICP inductor—1150 W. The following reagents were used for sample preparation: concentrated hydrochloric and nitric acids of ultrapure grades (state standard specification 11125-84, 14261-77), deionized water purified with the Direct-Q3 system (Millipore) > 18 MΩ/cm, high-purity argon, and standard solutions of gold (gold standard solution MSDS, 170216, Merck) and silver (silver standard solution, MSDS, 119797, Merck). The sample was dissolved with a mixture of concentrated hydrochloric and nitric acids (3:1). Disposable plastic tubes with a volume of 15 and 50 mL, a polypropylene container with a volume of 20 mL, and an automatic pipette of variable volume (1.00–5.00 mL, 100–1000 µL, and 10–100 µL) were used for the sample preparation. The most intense analytical lines (without spectral interference) 208.209, 242.795, and 267.595 nm, and 328.068 and 338.289 nm were used for the determination of Au and Ag, respectively. The data accuracy was confirmed by the spike experiment.

### 2.6. Electrochemical Characteristics of Ir and Pt Films

The properties of the corrosion resistance of Ti-alloy substrate, Ir, and Pt films were studied by the method of anodic polarization using a potentiostat–galvanostat P-30J (Elins, Russia). A 3.5 wt.% aqueous solution of NaCl was used as an electrolyte. A standard three-electrode cell with a saturated silver chloride electrode as a reference electrode and Pt electrode (size of 15 × 15 × 0.5 mm^3^) as a counter electrode was used for electrochemical investigations. The potentiodynamic current–voltage curves were recorded at a sweep rate of 20 mV/min. All experiments were performed at room temperature (25 °C).

### 2.7. Biological Research

#### 2.7.1. Preparation of the Experimental Samples

Before the biological tests, the experimental samples were sterilized by gas ethylene oxide treatment in a gas sterilizer Steri-Vac 5XL (3M^TM^, Maplewood, MI, USA). The concentration of ethylene oxide was 750 mg/L, the temperature in the chamber was 37 °C, the humidity was 70%, and the sterilization time was 3 h. Aeration was carried out at a sterilization temperature of at least 8 h. Pre-sterilization treatment was as follows: 30 min exposure in 96% ethanol and washing in sterilized water for 30 min.

#### 2.7.2. Preparation of the Mononuclear Blood Cells

Peripherical blood mononuclear cells (PBMC) were isolated from the peripheral blood of three healthy donors after receiving informed consent. First, 3 mL of whole EDTA-stabilized blood was diluted with a sterile phosphate-buffered saline (PBS, pH 7.2) in a ratio of 1:1; the diluted blood was layered on a density gradient medium Limpholyte^®^-H (Cedarlane, ON, Canada) and centrifuged at 400× *g* for 20 min. The isolated PBMCs were washed three times with phosphate buffer, followed by centrifugation. The number of living cells was determined using a cell counter Countess II (ThermoFisher Scientific, Waltham, MA, USA).

#### 2.7.3. Determination of Cytotoxicity of Samples by the XTT Test

Washed PBMCs were cultured with experimental samples in a 24-well plate at a concentration of 7.5 × 10^5^ cells/well in 1 mL of the culture medium RPMI-1640 containing 10% fetal bovine serum (Thermo Fisher Scientific, Waltham, MA, USA), 100 U/mL of penicillin, 100 U/mL of streptomycin, and 2 mmol/L of l-glutamine in a CO_2_ incubator. Intact PBMCs in the culture medium were used as a control. After 24 and 48 h, the experimental samples were removed, and the viability of PBMC was determined using the XTT cell proliferation assessment kit (Applichem, Barcelona, Spain) and the iMark Microplate Absorbance Reader (Bio-Rad, Hercules, CA, USA) according to the manufacturer’s recommendations. The results were presented as a percentage of the control value.

#### 2.7.4. Investigation of the Cytokine Composition of the PBMC Culture Medium

Five hundred microliter samples of the culture medium were taken at the previous stage immediately before the XTT test. PBMC secretion of cytokines and other factors (GM-CSF, IFNγ, IL-1b, IL-2, IL-4, IL-5, IL-6, IL-7, IL-8, IL-10, IL-12 (p70), IL-13, IL-17A, IL-21, IL-23, Fractalkine, I-TAC, MIP-1a, MIP-1b, MIP-3a, and TNF-a) was studied using a 21-plex Human High-Sensitivity T-cell Panel (Merck-Millipore, Burlington, MA, USA) by automatic flow luminometer Luminex 200 according to the manufacturer’s recommendations.

#### 2.7.5. Studies of the Antibacterial Activity of Samples

For the study of antibacterial activity, standardized pure cultures of *S. epidermidis*, *S. aureus*, *Str. pyogenes*, *P. aeruginosa*, and *Ent. faecium* were plated with a loop on a Petri dish in the appropriate culture medium. A sterile experimental sample (a titanium disc with experimental coating) was placed in the first quadrant of the dish. Standard discs impregnated with antibiotics specific to this bacterial culture were placed in the second, third, and fourth quadrants of the dish. Immediately after application of the discs, Petri dishes were placed in a thermostat and incubated for 1 day at 35 °C. The result was photodocumented using GelDoc XR. The diameter of the growth retardation zones was measured using the Quantity One software (Bio-Rad, Hercules, CA, USA).

#### 2.7.6. Statistical Analysis

Data were expressed as means ± standard deviation (SD). The statistical analysis and significance were evaluated using a one-way analysis of variance (ANOVA) followed by a post hoc Tukey test. All analyses were performed using a Statistica 13 software (TIBCO Software, Palo Alto, CA, USA). Differences between the control and treated samples were considered significant at *p* < 0.05.

#### 2.7.7. Valuation of Sample Biocompatibility in Histological Studies

This study was approved by the Local Ethics Committee of the E. Meshalkin National Medical Research Center of the Ministry of Health of the Russian Federation. All parts of the protocol were performed in accordance with the recommendations for proper use and care of laboratory animals (European Communities Council Directive 86/609/CEE) and the principles of the Declaration of Helsinki. Experimental samples (11 pairs) were implanted subcutaneously of Wistar rats weighing 150–200 g. Animals were anesthetized with an intraperitoneal injection of 50 mg/kg Zoletil-100 (Virbac, Carros, France), the hair on the back was shaved, and the skin was treated with betadine. Three or four experimental samples were placed in preformed subcutaneous pockets on both sides of the back. The skin incision was sutured with a Prolen 5/0 (B.Braun, Bethlehem, PA, USA) and treated with betadine. For histological examination of post-implantation inflammatory reactions, the samples were removed after 1 and 3 months together with surrounding tissues, fixed in 10% buffered formalin for 24 h; metal implants were retrieved, and tissue paraffin blocks were prepared according to the standard histological procedure. Five micrometer sections were made using HM340 microtome (Microm, Dreieich, Germany) and stained with hematoxylin–eosin. The morphological studies and micrometry of the samples were performed using an optical microscope Axioskop 40FL with an AxioVision v. 4.7 software (Carl Zeiss, Jena, Germany). The lymphocyte infiltration scoring scheme was as follows: 0, no sign of inflammation; 1, mild inflammation; 2, moderate inflammation; 3, severe inflammation. The thickness of the fibrous capsule surrounding the sample was estimated as the average value of 10 measurements made uniformly along the entire perimeter of the sample.

## 3. Results and Discussion

### 3.1. Characterization of Ir, Pt, and PtIr Coatings

Ir, Pt, and PtIr coatings were deposited on both sides of Ti-alloy substrates by an MOCVD method at the previously determined experimental parameters [31,37] in order to obtain metal coatings without impurities of oxide phases with a thickness of more than 1 µm. The ratio of metals in bimetallic samples was set as 1:1.

#### 3.1.1. Iridium Coatings

**Figure 1:**
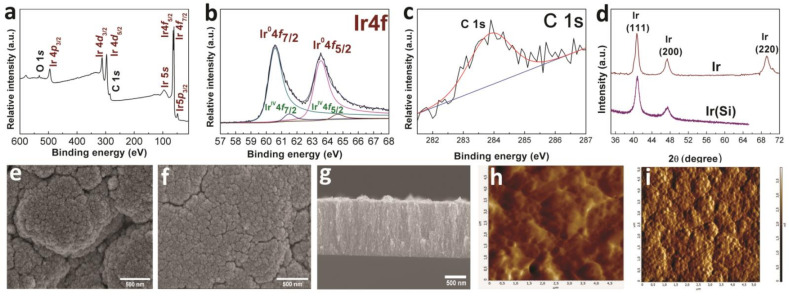
XPS spectra of Ir(Si) sample after etching with Ar^+^ (**a**), fitting of Ir 4*f* spectra (**b**), and fitting of C1*s* spectra (**c**); XRD patterns of Ir and Ir(Si) samples (**d**); SEM micrographs of the sample surface (Ir (**e**) and Ir(Si) (**f**)) and cross-section of Ir(Si) sample (**g**); AFM micrographs of Ti-alloy without (**h**) and with Ir coating (**i**).

According to the XPS data, the Ir(Si) sample after etching (Figure 1a) had the following composition: Ir 87, C 9, O 4 (at.%). Iridium was present mainly in the metallic form Ir^0^ (binding energy for Ir^0^ 4f_7/2_ is 60.4 eV) with an admixture of the oxidized form of iridium (binding energy for Ir^IV^ 4f_7/2_ is 63.2 eV) (Figure 1b). The ratio of 4f_7/2_ peak areas of Ir^0^:Ir^IV^ was 1:0.02, i.e., the content of oxidized iridium was approximately 2% of the total amount. The analysis of the position of C1*s* peaks after etching showed that carbon was present in the form of *sp*^2^-hybridized amorphous graphite (283.9 eV) (Figure 1c). According to the XRD data (Figure 1d), the main crystal phase recorded in the Ir and Ir(Si) samples was FCC-Ir (2θ = 40.8(111), 46.7(200), 68.8(220), JCPDS Card No.: 000-46-1044), while the IrO_2_ phase in the samples was not recorded within the method error. The XRD pattern of the Ir sample showed low-intensity reflections from the Ti-alloy substrate. Thus, the content of the IrO_2_ phase in the Ir(Si) and Ir samples was relatively small. The crystallite sizes calculated according to the Debye–Scherrer equation were 12 ± 1 nm and 15 ± 2 nm in the Ir(Si) and Ir samples, respectively. According to SEM data (Figure 1e,f), the surfaces of the Ir(Si) and Ir samples were porous with globule-like agglomerates (0.1–1 µm) and voids (0.01–0.1 µm). The sizes of agglomerates and voids were maximal for the Ir coating on a Ti-alloy substrate (Figure 1f). Iridium coatings had a characteristic dendritic columnar structure with the thickness on a silicon substrate of about 1.4 µm (Figure 1g).

According to AFM data, the initial surface of Ti alloy substrate is formed by disordered grains with dimensions of 0.05–0.5 µm with an average roughness of 84 nm (Figure 1h); after the deposition of an iridium coating (sample Ir), the roughness increased to 126 nm (Figure 1i).

#### 3.1.2. Platinum Coatings

**Figure 2:**
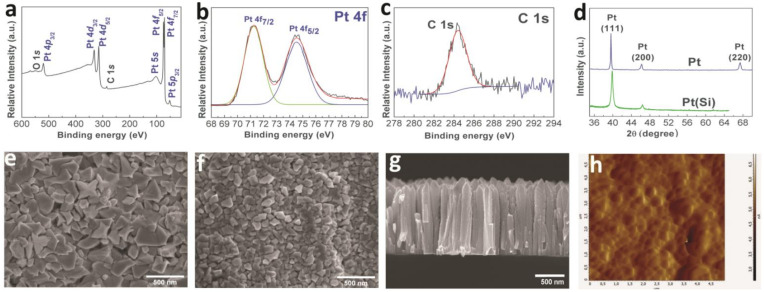
XPS spectra of Pt(Si) sample after etching with Ar^+^ (**a**), fitting of Pt 4*f* spectra (**b**), and fitting of C1*s* spectra (**c**); XRD patterns of Pt and Pt(Si) samples (**d**); SEM micrographs of the sample surface (Pt (**e**) and Pt(Si) (**f**)) and cross-section of Pt(Si) sample (**g**); AFM micrograph Pt sample (**h**).

According to the XPS data (Figure 2a), the Pt(Si) sample after etching had the following composition: Pt 89; C 11 (at.%); platinum was present in the metallic state Pt^0^ (binding energy for Pt4f_7/2_ is 71.2 eV) (Figure 2b). Similar to the iridium sample described above, carbon in the Pt(Si) sample was also present in the form of *sp*^2^-hybridized amorphous graphite (Figure 2c). According to the XRD data, the samples of Pt (Si) and Pt contained a single crystalline phase of FCC-Pt (2θ = 39.7(111), 45.5(200), 67.2(220), JCPDS Card No.: 010-87-0647) (Figure 2d); the calculated crystallite sizes were 69 ± 7 nm and 82 ± 8 nm, respectively.

According to the SEM data (Figure 2e,f), surfaces of the Pt(Si) and Pt samples were formed by pyramidal agglomerates tightly adjacent to each other. The agglomerate sizes were 0.15–0.2 and 0.3–0.5 µm for the Pt(Si) samples (Figure 2e) and Pt (Figure 2f), respectively. An increased size of the surface agglomerates in the Pt sample in comparison with the Pt(Si) sample may indicate higher growth rates of Pt films on metal (Ti-alloy) surfaces [31]. The Pt films had a columnar dagger-like structure with a thickness of ~1.4 µm (sample Pt(Si)) (Figure 2g). Pt films were characterized by a minimum roughness of 69 nm (Figure 2h).

#### 3.1.3. Bimetallic Coatings

**Figure 3:**
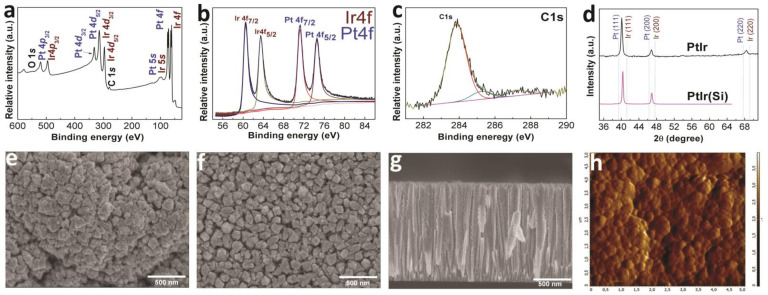
XPS spectra of PtIr(Si) sample after etching with Ar^+^ (**a**), fitting of Pt 4*f* and Ir 4*f* spectra (**b**), and fitting of C1*s* spectra (**c**); XRD patterns of PtIr and PtIr(Si) samples (**d**); SEM micrographs of the sample surface (PtIr (**e**) and PtIr(Si) (**f**)) and cross-section of PtIr(Si) sample (**g**); AFM micrograph PtIr(Si) sample (**h**).

According to the XPS data (Figure 3a), the PtIr(Si) sample after etching had the following composition: Ir 51; Pt 41; C 7; O 2 (at.%), which is close to the experimentally specified metal ratio (1:1). The positions of the Ir and Pt 4*f*_7/2_ peaks at 61.1 and 71.2 eV in the XPS of the PtIr coating indicated the presence of both elements in the metallic state in the sample (Figure 3b). The analysis of the C1*s* peak positions after the sample etching showed that carbon was present in the form of *sp*^2^-hybridized amorphous graphite (284.1 eV), as well as in the form of C-O (285.2 eV) and C=O (287.5 eV) species (Figure 3c).

According to the XRD data, the PtIr and PtIr(Si) samples were formed by a solid solution based on FCC metals (Figure 3d). The ratio of metals in the samples estimated according to the Vegard’s model was Pt_0.45_Ir_0.55_ (lattice parameter a = 3.867 ± 0.004 Å), which correlates with the ratio of metals determined by the XPS method. The XRD pattern of the PtIr sample showed low-intensity reflections from the Ti-alloy substrate. According to the XRD data, the calculated crystallite sizes were 28 ± 3 nm and 32 ± 3 nm in the PtIr(Si) and PtIr samples, respectively. According to the SEM data (Figure 3e,f), similarly to the Pt and Pt(Si) samples, the surfaces of the PtIr(Si) and PtIr samples were formed by pyramidal agglomerates; however, these agglomerates were not located densely, but with the presence of voids as in the case of the Ir(Si) and Ir samples. The dimensions of the surface agglomerates were 0.08–0.1 and 0.15–0.25 µm for the PtIr(Si) samples (Figure 3e) and PtIr (Figure 3f), respectively. The PtIr(Si) sample was characterized by a columnar dendritic structure [37]. The thickness of PtIr(Si) films was ~1.5 µm (Figure 3g). The PtIr samples were characterized by a roughness value of 96 nm, i.e., they occupied an intermediate position between the Ir and Pt coatings (Figure 3h).

#### 3.1.4. Corrosion Resistance Test of Coatings

**Figure 4:**
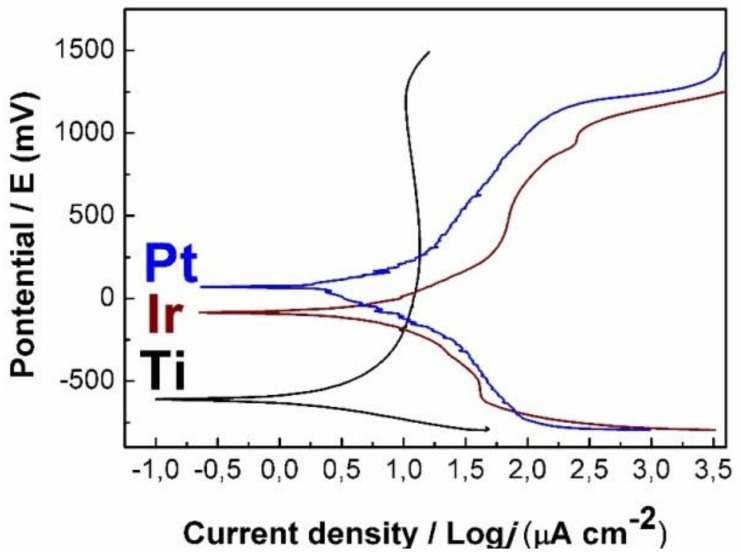
Polarization curves of Ir and Pt samples in comparison with uncoated Ti-alloy.

The corrosion resistance of the samples in biological solution was investigated using the electrochemical method. Figure 4 shows the polarization curves of a Ti-alloy in comparison with the Pt and Ir samples. The character of the polarization curve of Ti-alloy showed that the oxidation of its surface began at low currents (0.1 µA·cm^−2^). The rapid growth of the potential at a current density above 8 µA·cm^−2^ indicated the formation of a passivating TiO_2_ layer on the surface [38]. At the same time, in the case of Pt and Ir samples, electrochemical processes occurred at the higher current densities (0.25–0.29 µA·cm^−2^), indicating their increased corrosion resistance. The potentials (E_corr_) determined from the graph were 64, −87, and −608 mV for the Pt, Ir, and Ti-alloy samples, respectively. Thus, the Pt sample demonstrated the highest E_corr_ potential and, as a consequence, the highest corrosion resistance among the studied samples.

### 3.2. Characterization of Noble-Metal Coatings Modified by Au and Ag Nanoparticles

**Figure 5:**
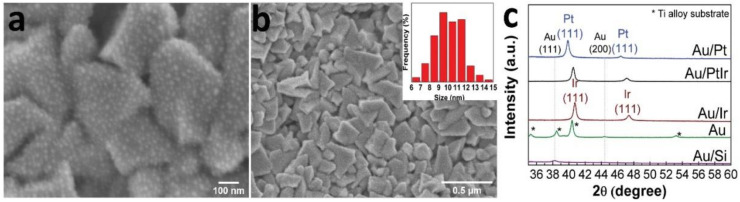
SEM micrographs of the surface of Au/Pt sample (**a,b**) with AuNP size distribution, and XRD patterns of the series of Au/Si, Au, Au/Ir, Au/Pt, and Au/PtIr samples (**c**).

Gold nanoparticles (AuNPs) were deposited by a pulsed-MOCVD method (10 cycles) onto the metal surfaces (Au, Au/Ir, Au/Pt, and Au/PtIr samples) and Si(100) substrates (Au(Si)). Typical microphotographs of AuNPs are shown in Figure 5 using the Au/Pt sample as an example. The surface of the Au/Pt samples was formed by irregularly shaped nanoparticles (size 10 ± 1 nm), which were evenly distributed on the surface agglomerates of platinum. Reflexes related to Au were not visualized on the XRD patterns of the Au/Ir, Au/Pt, and Au/PtIr samples apparently due to their low concentration. According to the XRD data, the calculated AuNPs sizes in the Au(Si) and Au samples were 14 and 11 nm, respectively. The Au content in the samples of Au(Si), Au, Au/Ir, Au/Pt, and Au/PtIr was 2–3 μg/cm^2^.

**Figure 6:**
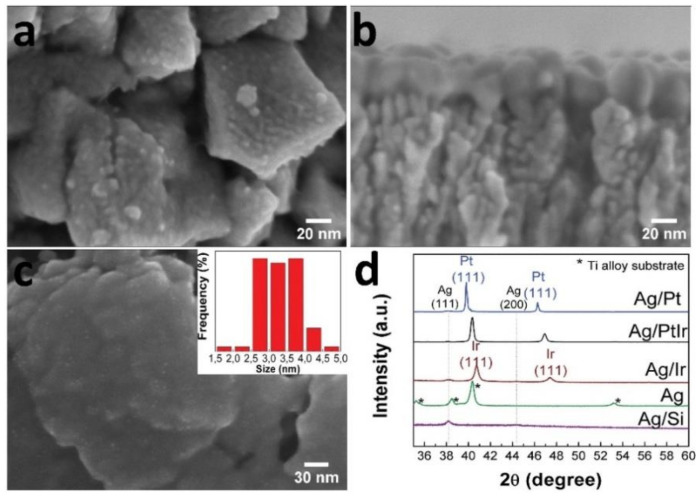
SEM micrographs of the surface of Ag/Pt sample (**a**) and cross-section of Ag/Ir sample (**b**), surface of Ag sample on Ti-alloy with AgNPs size distribution (**c**), and XRD patterns of series of Ag/Si, Ag, Ag/Ir, Ag/Pt, and Ag/PtIr samples (**d**).

The surface of Si(100) and metal samples (Ag, Ag/Ir, Ag/Pt, and Ag/PtIr) was modified with metallic Ag by PVD. Discontinuous Ag coatings (35–40 nm) consisting of small particles (10–20 nm) and clusters were formed on the following surfaces: Ag/Ir, Ag/Pt, and Ag/PtIr (Figure 6a,b). Discontinuous coatings with ultradispersed AgNPs with sizes up to 5 nm were formed on the surface of the Ti-alloy (Figure 6c). According to the XRD data, the calculated Ag particle sizes in the Ag(Si), Ag/Ir, Ag/Pt, and Ag/PtIr samples were 20–32 nm (Figure 6d). The silver content in the Ag/Ir, Ag/Pt, and Ag/PtIr samples was 26–32 µg/cm^2^, while it was 8–9 µg/cm^2^ in the Ag sample on the Ti-alloy disc.

### 3.3. Biological Studies

#### 3.3.1. Cytotoxic Activity

**Figure 7:**
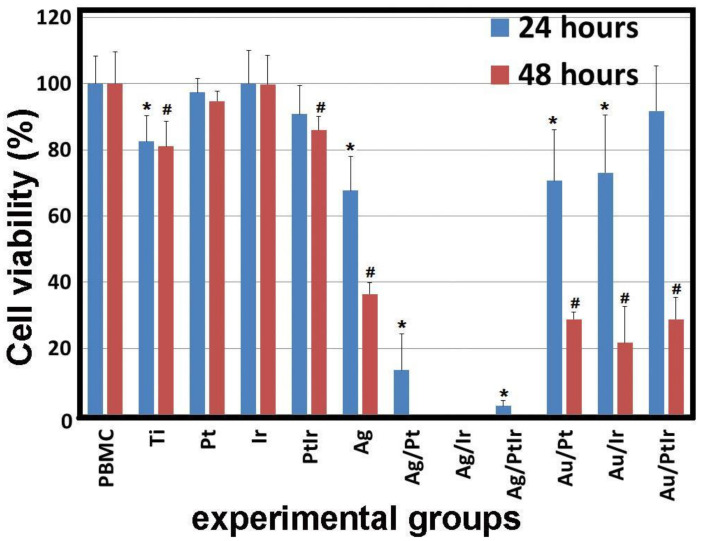
Viability of PBMC human cells after 24 and 48 h of cultivation in the presence of series samples (Ti-alloy with various combinations of metals on films); * *p* < 0.05 compared with control (for groups of 24 h), ^#^ *p* < 0.05 compared to control (for groups of 48 h).

All the studied samples were divided into several experimental groups: Ti-alloy (Ti), Ti-alloy with monophase noble-metal coatings (Pt, Ir, and PtIr), Ti-alloy with monophase Ag coating (Ag), Ti-alloy with heterophase noble-metal coatings containing AuNPs (Au/Pt, Au/Ir, and Au/PtIr), and Ti with heterophase noble-metal coatings containing Ag layers (Ag/Pt, Ag/Ir, Ag/PtIr). The samples of various mono- and heterostructures were shown to have different cytotoxic activity. The samples from the It, Pt, and PtIr groups (three in each group) had the least effect on PBMC after 24 and 48 h of cultivation. In other groups, a significant decrease in cell viability compared to the control (PBMC) group was observed (Figure 7).

The thin Ag film on the Ti disc, as well as the pure Ti-alloy, had more pronounced activity; cell viability decreased to 67.4% ± 10.3% and 82.5% ± 7.8%, after 24 h, respectively, and to 36.4% ± 3.6% and 80.9% ± 7.7% after 48 h. These results are consistent with the data of other researchers who evaluated the cytotoxic effect of silver ions and nanoparticles on human cells [39,40,41] and found that the cytotoxic effect of nanoparticles, microparticles, and silver ions on osteoblasts and osteoclasts correlated with the antibacterial efficacy against *Staphylococcus epidermidis*; silver nanoparticles (50 nm) showed a strong cytotoxic effect [39]. Weak cytotoxic effects were observed for silver microparticles (3 µm). Greulich and coauthors [41] showed that the toxic effect of silver on *Escherichia coli, Staphylococcus aureus*, human mesenchymal stem cells (hMSC), and PBMCs was manifested in a similar concentration range. The effective toxic concentration of silver in relation to bacteria and human cells was almost the same.

The most toxic samples were Ag/Pt, Ag/Ir and Ag/PtIr, in which silver was deposited on the films of platinum group metals. A similar result was obtained by Abuayyash and coauthors [15] in their study of the antibacterial properties of Ag/Pt, Ag/Ir, and Ag/Au structures. Since noble metals are characterized by low electrochemical activity (standard electrochemical potentials Pt/Pt^2+^ = 0.963 V; Ir/Ir^3+^ = 1.156 V, Au/Au^3+^ = 1.498 V [42]), the process of anodic dissolution of silver as the more active metal (Ag/Ag^+^ = 0.7996 V) was observed in these galvanic pairs. Thus, silver ions were released into the solution more efficiently than in the case of the Ag/Ti structure, where silver was a cathode and, therefore, the electrochemical activation of the process did not occur. After 24 h, the observed toxic effect correlated with the potential difference in pairs; the highest viability was observed in the Ag/Pt group (13.4% ± 10.9%); the viability was lower for Ag/PtIr (2.5 ± 1.6%), and, in the case of Ag/Ir, complete cell death was observed. After 48 h, no viable cells remained for all samples from the experimental group under consideration (Ag/Pt, Ag/Ir, and Ag/PtIr), but this was not achieved for other groups.

Comparison of the samples containing gold nanoparticles (Au/Pt, Au/Ir, and Au/PtIr) with other experimental groups after 24 h of cocultivation with PBMCs showed that the cell viability with Au/Pt (70.6% ± 15.5%) and Au/Ir (73.0% ± 17.5%) samples was lower than platinum metal samples (Pt: 97.3% ± 4.2%; Ir: 100% ± 9.9%; PtIr: 90.8% ± 8.6%) and did not significantly differ from the Ti-alloy groups (82.5% ± 7.8%) and Ag (67.6% ± 10.3%), but was higher than silver on platinum metals. It is noteworthy that the cell viability in the Au/PtIr group (91.7% ± 17.5%) after 24 h did not differ from the groups with platinum metals (Pt, Ir, and PtIr) and was higher than in the experimental groups containing Ag. However, after 48 h, the viability of PBMC in the Au/Pt, Au/Ir, and Au/PtIr groups decreased to 28.8% ± 2.2%, 21.7% ± 11.0%, and 28.8% ± 6.7%, respectively, and was lower than in platinum metal groups (Pt, Ir, and PtIr) and in the Ti group. Shareena Dasari et al. [43] partially confirmed our results that gold nanoparticles reduce the viability of PBMCs and explained it by the presence of Au^+^ and Au^3+^ ions in unpurified synthetic gold nanoparticles, which can enhance the antibacterial properties and, as a result, affect the survival of human cells. At the same time, the cause of the cytotoxic effect of gold nanoparticles has not been definitively clarified. It is suggested that the particle size is one of the key parameters determining the biological effect of AuNPs, and the importance of nanometer size in biological effects has been emphasized, even for inert materials such as gold [44,45].

In particular, many in vitro experiments showed no noticeable toxicity of AuNPs with a size of about 100 nm, provided that the upper limit of the applied dose did not exceed a value of about 10^12^ particles/mL. In contrast, AuNPs with a diameter from 1 to 2 nm have a potentially high toxicity due to the possibility of irreversible binding to key biopolymers, e.g., to one of the forms of DNA [44,45]. However, in our work, larger particles (10–14 nm) were used. Mironova and coauthors [46] showed that Au particles with sizes of 13 and 45 nm penetrate neither the nucleus nor the mitochondria (for 6 days), but are accumulated by large vacuoles in the cell cytoplasm.

It is worth noting that the AuNPs are mainly obtained using the method of “wet chemical synthesis”, which involves functionalization by organic molecules, followed by the study of the localization and influence on all human organs. In our work, AuNPs were obtained by the gas-phase method without the presence of additional organic and ionic fragments. Therefore, we showed the effect of toxicity of metal nanoparticles; however, the aspect of their possible diffusion into the cytoplasm of the cell needs further investigation. An alternative hypothesis that could explain the cytotoxic effect of AuNPs is an activity in generating ROS fragments that cause oxidative cell stress [2]. Most studies attribute the ability to generate ROS to ultradispersed Au particles (several nanometers). However, some studies showed the possibility of the abovementioned properties in particles of the same size as those described herein. For example, Piano and coauthors [47,48] showed that the toxicity of 7–20 nm AuNPs to dermal fibroblasts is due to oxidative cell stress. Lastly, some researchers suggested the presence of a synergistic antimicrobial and cytotoxic effect of metal binary composites [49,50]. For example, Li et al. [49] showed that AgNPs were more toxic than AuNPs, whereas the introduction of gold into silver nanoparticles could diminish their environmental impact by reducing the amount of bioavailable Ag. Formaggio and coauthors [50] examined the toxicity of bimetallic AuPt nanoparticles in vitro and in vivo and evaluated the antimicrobial activity compared to AuNPs and AgNPs. Bimetallic particles showed increased antimicrobial activity compared to AuNPs, but lower activity compared to AgNPs. Apparently, the synergistic effect also contributed to a decrease in cytotoxicity in the Au/Pt, Au/Ir, and Au/PtIr groups.

#### 3.3.2. Cytokine Release by PBMC

**Figure 8:**
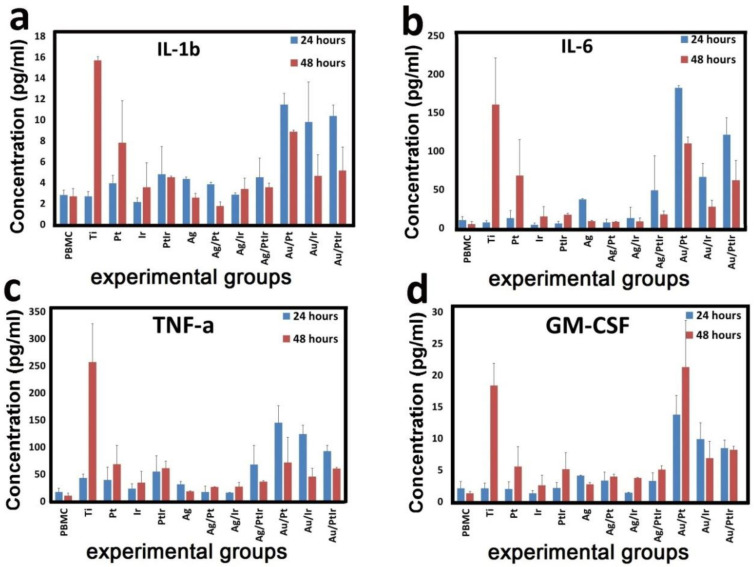
Concentration of dissolved cytokines IL-1b (**a**), IL-6 (**b**), TNF-a (**c**), and GM-CSF (**d**) in the culture medium after cocultivation with PBMCs and a series of experimental samples for 24 and 48 h.

The study of the immunomodulatory potential of samples with various variants of monophase noble-metal coatings and heterostructures was performed by analyzing the secretion of cytokines and growth factors of human PBMCs. A significant increase in the concentration of the main proinflammatory cytokines IL-1b, IL-6, and TNF-a, as well as factor GM-CSF, was found (Figure 8).

Pure Ti-alloy and Pt-coated samples caused delayed cytokine secretion of PBMCs. The concentration of cytokines in the supernatant after 24 h did not differ significantly from the control group, but increased sharply after 48 h of PBMC cultivation. The concentration of IL-1b, IL-6, TNF-a, and GM-CSF with Ti-alloy samples increased 5.9-, 33.1-, 24.8-, and 13.8-fold, respectively, while that with Ir samples increased only 1.3-, 14-, 6.6-, and 4.2-fold. Our studies of Ti-alloy samples partially correlate with data obtained by Cachinho et al. [51], who studied the release of cytokines in a mixed culture of MNCs in response to stimulation with titanium, stainless steel, and other alloy samples. They experimentally proved that metal nanoparticles induce an IL-6 and IL-1 response, but not a TNF-α response.

At the same time, experimental samples with gold nanoparticles (Au/Pt, Au/Ir, and Au/PtIr) cocultured with PBMCs caused an increase in cytokine secretion after both 24 and 48 h. The most pronounced changes were observed in the Au/Ir group, where the concentration of IL-1b, IL-6, TNF-a, and GM-CSF increased 4.1-, 17.6-, 8.6-, and 6.5-fold after 24 h and 3.3-, 12.4-, 6.9-, and 12.3-fold after 48 h, respectively. In other groups of gold-containing samples, changes in cytokine secretion had the same tendency (differences with the control group were significant).

Some anti-inflammatory and toxic properties of the gold complex were described earlier [52], but that complex induced Au^+^ ions with increased toxicity to immune cells. On the contrary, our data indicated the activation of lymphocytes and the release of proinflammatory cytokines. Perhaps, this was a manifestation of a nonspecific immune response of the PBMCs and the absence of gold ions. Other experimental groups caused much smaller changes in cytokines release. The differences between these groups and the control group were unreliable, except for the PtIr, Ag, and Ag/PtIr groups [53].

Other cytokines (IL-2, IL-5, IL-7, IL-8, IL-10, IL-12, IL-13, and IL-21) were absent in the samples, or their concentration was lower than the minimum sensitivity of test. This fact confirmed the absence of anti-inflammatory properties of the experimental samples.

The immunomodulatory effect of implantable materials is important in the treatment of cancer, rheumatic diseases, and other immunological disorders. However, the newly developed biomaterials and medical implants must have a high level of bioinertness to ensure the safety of their further medical application. In acute in vitro immunotoxicity tests of Ti-alloy and Au/PtIr samples, we showed the highest level of secretion of IL-1b, IL-6, TNF-a, and GM-CSF cytokines, which are responsible for the development of post-implantation inflammation, lymphocytic infiltration, and fibrosis [54]. Further histological studies will confirm this fact.

#### 3.3.3. Studies of Antibacterial Properties

**Figure 9:**
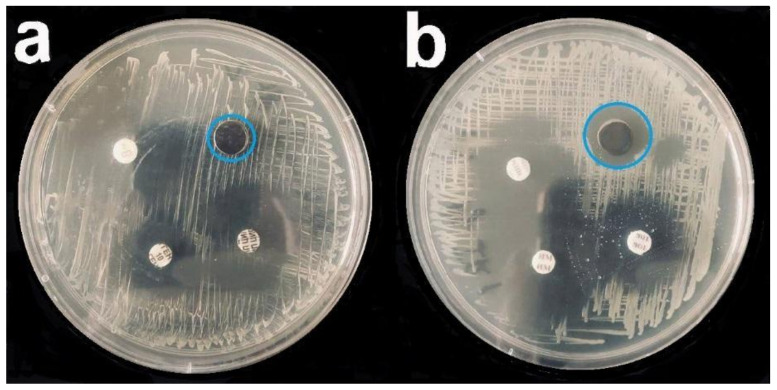
Bacterial growth inhibition assay (*P. aeruginosa*) for Ir (**a**) and Ag/Ir (**b**) samples; blue circle—zone of inhibition. The remaining samples were standard discs with control antibiotics (cefepime, imipenem, and tobramycin).

Studies of antibacterial properties using five cultures (*S. epidermidis, S. aureus, Str. pyogenes, P. aeruginosa*, and *Ent. faecium*) showed that silver-containing coatings were the best in inhibiting bacterial growth (Figure 9, Table 1).

In comparison with the control titanium-alloy samples, the diameter of the bacterial growth inhibition zone of silver samples was 1.3–2-fold larger, depending on the group (Figure 9). Antibacterial properties of the materials were investigated using the most common nosocomial and hospital pathogens. Some of our results were confirmed by the studies of Ramstedt M. (2009) [55] and Greulich S. (2012) [41]. They found that an antibacterial effect persisted for some Gram-positive (*S. aureus*) and Gram-negative (*E. coli* and *P. aeruginosa*) bacterial cultures.

**Figure 10:**
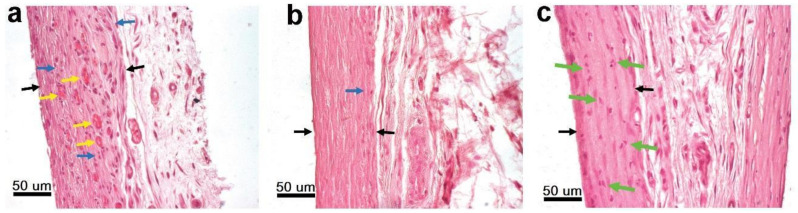
Reduction in lymphocytic infiltration and the formation of a connective tissue capsule around uncoated Ti-alloy after 1 month (**a**) and 3 months (**b**) of implantation. Destruction of lymphocyte nuclei in the fibrous capsule surrounding Ag/Pt samples (green arrows) (**c**). Black arrows—capsule border, blue arrows—accumulations of lymphocytes, yellow arrows—newly formed blood vessels.

The results of the antibacterial activity of Ag and platinum metal heterometallic systems against other Gram-positive microorganisms (*S. epidermidis, Str. pyogenes,* and *Ent. faecium*) were obtained in this study for the first time. The mechanism of the silver ion antibacterial action was investigated in previous works [56]. According to these studies, an increase in the intracellular concentration of Ag^+^ leads to an increase in free radicals, activation of the oxidative stress mechanism, and launch of programmed cell death [57]. In our study, Ag, Ag/Ir, and Ag/PtIr samples demonstrated the highest antibacterial activity (Figure 10). In the first sample, this may have been due to the noticeably smaller Ag particle sizes formed on the Ti-alloy substrate, compared to the coatings on noble metals (Section 3.2). The more pronounced activity of Ag/Ir and Ag/PtIr compared to Ag/Pt is correlated with a higher potentials difference, which should contribute to a more efficient leaching of silver ions [15].

At the same time, the ZOI size for all samples with gold nanoparticles on the platinum metal coatings was practically the same as for uncoated Ti-alloy substrates. The absence of the expected antibacterial effect of gold nanoparticles may have been due to the insufficient duration of bacterial growth inhibition assay, as noted in previous reviews [44,45], since we observed a toxic effect on PBMCs only after 48 h (Figure 7). Thus, long-term studies of the antibacterial effect of samples of gold nanoparticles are a necessary stage for further research.

#### 3.3.4. Histological Studies

##### Structural Features of the Fibrous Capsule

Lymphocytic infiltration of the fibrous capsule (FC) around the implanted samples as a characteristic reaction of the body to a foreign body was noted for the Ti, Ir, Ag, Ag/Pt, Au/Pt, and Au/PtIr groups after 1 month [58]. The most pronounced reaction was observed in the group of Ti-alloy samples (3 points), whereas a medium-intensity reaction was observed in the case of Ir and Au/Pt groups (1–2 points for each) and the Ag and Ag/Pt groups (1 point for each) (Figure 10). The least intense reaction (0–1 point) was observed in the Au/PtIr sample group.

In Ti, Ir, Ag, Ag/Pt, Au/Pt, and Au/PtIr groups of samples, except for the Au/Pt group, increased FC vascularization was observed (Figure 10a), with the blood vessels mainly located in the middle of the capsule. In other groups, neither lymphocytic infiltration nor increased FC vascularization was detected, indicating the completion of the active phase of foreign body encapsulation.

Fixing the processes of destruction of cell nuclei in the fibrous capsule of the Ag/Pt group after 1 month was an interesting observation (Figure 10c). This was an additional confirmation of the high cytotoxicity of this composition, which was previously detected in an in vitro test. However, for the Ag/Ir and Ag/PtIr groups, which also exhibited a high biocidal effect, nuclear destruction was not observed. There seems to be a contradiction here. However, it should be noted that, for the Ag/Ir and Ag/PtIr groups, the healing process was mostly finished, since lymphocytic infiltration of FC was no longer observed. A possible course is that the metals in Ag/Ir and Ag/PtIr samples had a higher potential difference between the cathode and the anode than in the case of the Ag/Pt; hence, the silver ion leaching was faster/more active.

In addition, the expressed morphology of Ir and PtIr coatings (roughness values of 126 and 96 nm) in comparison with Pt coatings (roughness values of 69 nm) could also contribute to a more intense of silver release from the surface of these samples. Therefore, in the case of Ag/Ir and Ag/PtIr samples, after 1 month of implantation, silver was already leached in the necessary concentration to have an active toxic effect, and the cells were already eliminated from the FC. In the case of the Ag/Pt group, the dissolution of silver proceeded more slowly; accordingly, we still observed the active influence of silver. The results of a quantitative study of Ag/Pt samples after implantation using ICP-OES showed that the maximum dissolution of silver (70–80%) occurred within 1 month, and it remained on the surface at a concentration of 5.5–8.9 µg/cm^2^. Furthermore, the process slowed down significantly; only 3.8–4.5 µg/cm^2^ of silver was found on the surface after 3 months of implantation. This process can be explained by the surface encapsulation of this coating and particles. One of the reasons may have been the formation of a layer of a poorly soluble silver compound with components of the biological environment, most likely chloride ions. Silver ions were kept in too low concentrations for an obvious toxic effect and, therefore, the healing process of the implant stopped, whereby there was no lymphocytic infiltration or increased vascularization of the FC.

Histological analysis of the samples after 3 months of implantation revealed the presence of lymphocytic infiltration only in the control group Ti-alloy (Figure 10b). The process had minimal activity (0–1 point). This fact is proof of the latent toxicity of titanium, which is the known and most common cause of the development of late postoperative complications and operations for the replacement of titanium implants [59,60].

##### Average Thickness of the Fibrous Capsule

**Figure 11:**
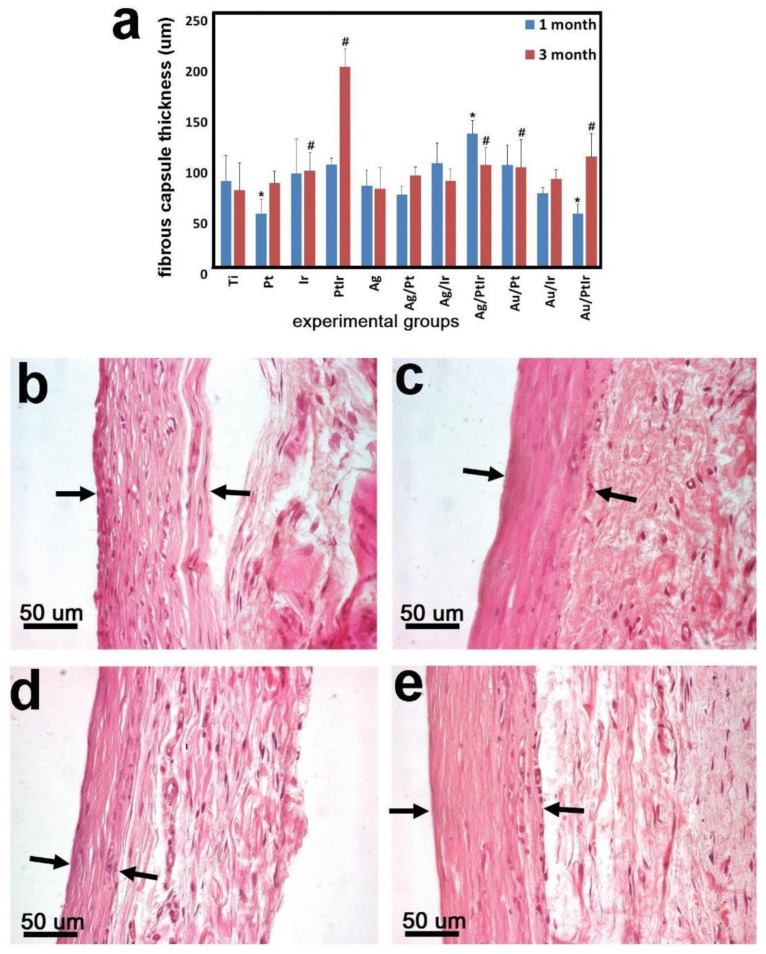
The thickness of the fibrous capsule around experimental samples after 1 and 3 months of subcutaneous implantation in rats (**a**); ^#^ significant differences with the control group (1 month), * significant differences with the control group (3 months). Dynamics of the formation of a fibrous capsule (black arrows—capsule border) around series of experimental samples with AgNPs: Au/Pt 1 month (**b**), Au/Pt 3 months (**c**), Au/PtIr 1 month (**d**), and Au/PtIr 3 months (**e**).

After 1 month of observation, significant differences with the control group (Ti-alloy samples) were shown for samples of the following groups: Pt (*p* = 0.0005), Ag/PtIr (*p* < 0.0001), and Au/PtIr (*p* = 0.0003). The FC thickness was 89.0 ± 25.4 μm (Ti), 51.9 ± 13.9 μm (Pt), 130.6 ± 13.0 µm (Ag/PtIr), and 51.9 ± 9.3 µm (Au/PtIr). No significant differences were noted for the other sample groups (Figure 11a).

After 3 months after implantation, the thickness of the FC changed slightly for almost all samples, indicating the stabilization of the encapsulation process. Two experimental groups of samples were an exception. In particular, a sharp increase in the FC thickness from 100.3 ± 6.1 μm (1 month) to 196.4 ± 18.1 μm (3 months) was found in the PtIr group after 3 months. A similar effect of approximately twofold growth was observed for the Au/PtIr group; the thickness increased from 51.9 ± 9.3 μm (1 month) to 107.9 ± 22.8 μm (3 months) (Figure 11). These results can be explained by the presence of the Pt–Ir electrochemical pair, which led to the anodic dissolution of platinum. Since both of these metals are chemically inactive, and the potential difference is small, the process was very slow, and the concentration of metal cations became sufficient for a biological response only after a long time. Such corrosion led to an increase in the cytotoxicity of the implant, which the organism continued to separate from. However, it should be taken into account that the thickness of the fibrous capsule in the Au/PtIr group after 1 month of implantation was almost twofold thinner, and, after 3 months, it did not significantly differ from the control Ti-alloy group (74.6 ± 26.9 μm).

The effect of a “delayed” increase in the thickness of the FC was not observed in the Ag/PtIr group. This was also consistent with the electrochemical approach. Firstly, silver leached as a more active metal, whereas the process of platinum leaching from the bimetallic coating began only after the complete dissolution of silver. That is why, for this group of samples, the growth of FC should be expected with longer implantation. The observed results correlated with recent work of Dalrymple et al. [61], which showed that Pt–Ir-coated steel electrodes became fibrotic during cochlear implantation and the capsule thickness slightly increased after 5 weeks of implantation. This was detected by an increase in the impedance, while no histological data were obtained. It was assumed that the process of metal cation leaching was activated under the action of the current during the chronic stimulation program. In addition, pinpoint losses of the PtIr coating on the electrodes and the presence of metal particles in the surrounding tissues were found. This may have happened due to electrochemical corrosion or damage of the coating during surgery. Stopping of the fibrous capsule growth for a long implantation period (more than 200 days) was also shown earlier for PtIr wire electrodes by the impedance measuring [62].

In general, histological differences in the structure of FC samples of the Ti-alloy implants and those coated with noble metals may have been the result of two different engraftment processes. Resorption of dead lymphocytes and macrophages in the Ti-alloy group 1 month after implantation led to the formation of a friable FC saturated with voids in the places of cell accumulation [63,64].

The maturation of FC by 3 months due to the thickening and compaction of connective tissue fibers synthesized by fibroblasts led to a decrease in their average thickness. Our results show that implants with noble metal coatings caused the formation of a dense fibrous capsule with a minimal level of immune response from the very beginning, which was consistent with the abovementioned data obtained for PtIr electrodes [61]. In this case, the increase in FC over time observed for samples with PtIr coating may have been due to the nonspecific toxic effect of Pt ions leaching from their surface, when the death of fibroblasts inside the capsule led to the start of the repair mechanism and the formation of a new layer of the fibrous capsule.

## 4. Conclusions

In this work, monophase (Ag, Ir, Pt, and PtIr) and heterophase (Ag/Pt, Ag/Ir, Ag/PtIr, Au/Pt, Au/Ir, and Au/PtIr) film materials were deposited on a biomaterial (medical alloy Ti_6_Al_4_V (Ti-alloy)) by gas-phase transportation methods to study their biological characteristics. Coatings of platinum group metals (Pt, Ir, and PtIr) with a thickness of 1.4–1.5 µm were deposited using an MOCVD method as bottom layers of film heterometallic film materials. It was shown that the corrosion resistance of Ti-alloy increased with the deposition of Pt and Ir coatings, and this effect was more pronounced for platinum due to a denser microstructure. Two types of antibacterial components, gold nanoparticles and discontinuous Ag coatings, were deposited on the surface of these coatings. AuNPs with a uniform distribution over the surface and dimensions of 11–14 nm were obtained using the pulsed-MOCVD method. The gold concentration on the surface of the Au/Pt, Au/Ir, and Au/PtIr samples was 2–3 µg/cm^2^. Discontinuous 35–40 nm Ag coatings were deposited by a PVD method. Such coatings of noble metals were made in the form of small-sized particles (10–20 nm) and their aggregates (clusters of 30–50 nm); the silver concentration was 26–32 μg/cm^2^. Ultradispersed AgNPs (up to 5 nm) with a silver content of 8–9 µg/cm^2^ were formed on the Ti surface.

Various in vitro studies including cytotoxic action and cytokine release by PBMCs (after 24 and 48 h of incubation) and antibacterial action against *S. epidermidis*, *S. aureus*, *Str. pyogenes*, *P. aeruginosa*, and *Ent. faecium* (ZOI diameter after 24 h) were performed for such noble-metal systems for the first time. The pattern of PMNC cytokine expression in response to Ti-alloy reflected the typical predominance of proinflammatory factors. It was shown that the deposition of Ir, Pt, and PtIr coatings reduced the cytotoxic effect of Ti-alloy (80–100% of the surviving cells versus 70%) and the production of cytokines. It should be noted that a similar cytokine profile was registered for AuNPs as for uncoated Ti-alloy. In addition, a “delayed” cytotoxic effect (after 48 h) was observed for all AuNP samples. This probably led to the absence of an antibacterial effect for this group after 24 h. Ag-containing film materials showed a cytotoxic action toward PBMCs and an antibacterial action toward all the strains used after 24 h. In the case of Ag/Ir and Ag/PtIr samples, the effect was the most pronounced and correlated with enhanced silver dissolution due to the “sacrificial” anode principle.

In vivo histological studies at 1 and 3 months after implantation in rats showed that the deposition of all types of noble-metal coatings led to an acceleration of the implant engraftment process compared to the uncoated Ti-alloy sample. Lymphocytic infiltration of the fibrous capsule was noted only for the Ti-alloy sample after 3 months, while the most pronounced reaction was registered after 1 month. This observation was consistent with the cytokine profile.

At the same time, the thickness of the fibrous capsule was comparable (70–100 μm) in most cases after 3 months; however, the mechanism of their formation was different for uncoated and coated Ti-alloy. A “delayed” (3 months) twofold increase in the fibrous capsule thickness was observed for PtIr and Au/PtIr samples, which may have been due to the slow dissolution of platinum as an anode in these galvanic pairs. During all implantation periods, there was a gradual dissolution of silver from surface, and the process slowed down over time. Presumably, this was due to the encapsulation of the surface because of the formation of a poorly soluble compound, e.g., AgCl.

Thus, this groundbreaking work represents the potential of application of heterophase coatings based on Au/Ag and corrosion-resistant platinum metals in the field of implantation and identifies many areas for further research.

## Figures and Tables

**Table 1:** Diameter of bacterial growth inhibition zone (mm).

Group of Samples	*P. aeruginosa*	*S. aureus*	*S. epidermidis*	*Str. pyogenes*	*Ent. gaecium*
Ti-alloy	10	10	10	10	10
Ag	19.9 ± 0.5	16.4 ± 0.5	20.3 ± 0.5	13.7 ± 0.3	16.0 ± 0.4
Ag/Pt	18.3 ± 0.9	16.0 ± 0.4	20.0 ± 0.3	14.0 ± 0.4	16.3 ± 0.6
Ag/Ir	20.1 ± 0.4	16.7 ± 0.4	20.0 ± 0.4	13.3 ± 0.5	16.0 ± 0.4
Ag/PtIr	19.5 ± 0.2	16.0 ± 0.3	20.3 ± 0.6	14.0 ± 0.5	16.3 ± 0.4

## Data Availability

The data presented in this study are available in this article or available on request from the corresponding author.

## Supplementary Materials

The following are available online at https://www.mdpi.com/article/10.3390/biomedicines9080851/s1. Synthesis and characterization of MOCVD precursors.
