# Supplementary material for: Noble Metals for Modern Implant Materials: MOCVD of Film Structures and Cytotoxical, Antibacterial, and Histological Studies
Source: Biomedicines. 2021 Jul 21;9(8):851. doi: 10.3390/biomedicines9080851 (PMC8389635; doi:10.3390/biomedicines9080851)
Supplement: Supplementary file 1 [file biomedicines-09-00851-s001.zip › biomedicines-1272187-supplementary.pdf]

# SUPPLEMENTARY INFORMATION

# Noble metals for modern implant materials: MOCVD of film structures, cytotoxical, antibacterial and histological studies

Svetlana I. Dorovskikh[1], Evgeniia S. Vikulova[1], Elena V. Chepeleva[2], Maria B. Vasilieva[2], Dmitriy A. Nasimov[2], Eugene A. Maksimovskii[1], Alphiya R. Tsygankova[1], Tamara V. Basova[1], Davis S. Sergeevichev[2], Natalya B. Morozova[1,*]

[1]Nikolaev Institute of Inorganic Chemistry SB RAS, 3 Lavrentiev Pr., Novosibirsk, 630090, Russia
[2]Meshalkin National medical research center» of the Ministry of Health of the Russian Federation, 15 Rechkunovskaya Str., Novosibirsk, 630055, Russia

## ***Section S1.*** *Synthesis and characterization of MOCVD precursors*

**[Ir(cod)(acac)]**. The complex was synthesized in the Schlenk apparatus (Scheme S1) by an interaction of 3.85 g (5.7 mmol) of $[Ir(cod)Cl]_2$ (97%, Sigma-Aldrich, CAS 12112-67-3) with 1.15 mL (11.5 mmol) of acetylacetone Hacac (99%, Dalchem, CAS 123-54-6) in 60 mL of diethyl ether with the addition of 1M water solution of KOH (11.5 mL, 11.5 mmol) during 12 hours at room temperature.

$$[Ir(cod)Cl]_2 \xrightarrow[Et_2O,\ Ar]{Hacac,\ KOH} [Ir(cod)(acac)]$$

**Scheme S1.** Scheme of the synthesis of [Ir(cod)(acac)].

The product was purified by gradient sublimation in vacuum (140°C, 5 $10^{-2}$ Torr); the yield of [Ir(cod)(acac)] was 95% (4.31 g, 10.8 mmol). The elemental analysis data (CARLO-ERBA-11008, mass %): for $C_{13}H_{19}O_2Ir$: found C, 39.3; H, 4.9, calc. C, 39.1; H, 4.8, melting point 155 °C.

**$[Pt(acac)_2]$**. The complex was synthesized by the interaction of 1 g (3.3 mmol) of $H_2[Pt(OH)_6]$ (99%, Sigma-Aldrich, CAS 51850-20-5) with 0.85 mL (8.5 mmol) of Hacac in 10 mL of 0.1M $HClO_4$ with an addition 0.12 mL (3.3 mmol) of HCOOH during 1 hour at 45 °C (Scheme S2).

$$H_2[Pt(OH)_6] \xrightarrow[HClO_4,\ HCOOH]{Hacac} [Pt(acac)_2]$$

**Scheme S2.** Scheme of the synthesis of $[Pt(acac)_2]$.

The product was purified by gradient sublimation in vacuum (170°C, 5 $10^{-2}$ Torr); the yield of $Pt(acac)_2$ was 50% (0.65 g, 1.7 mmol). The elemental analysis data (CARLO-ERBA-11008, mass %): for $C_{10}H_{14}O_2Pt$: found C 30.8; H 3.1; calc. C 30.5; H 3.6, melting point 240 °C.

**[$(CH_3)_2$Au(thd)]**. The complex was synthesized in two stages (Scheme S3) using 1 g (2.6 mmol) of K[$AuCl_4$] obtained as a precipitate during slow addition of 0.15 g (2.6 mmol) of KCl to an equimolar solution of H[$AuCl_4$] hydrate (metal content ≥ 47,8%, Krastsvetmet, CAS 16903-35-8). Firstly, 0.35 g (1 mmol) of [$(CH_3)_2$AuI]$_2$ was isolated with 40% yield as a result of the interaction of 1 g (2.6 mmol) of K[$AuCl_4$] and solution of $CH_3$MgI (10 mmol) in 200 mL of cooled diethyl ether in the Schlenk apparatus. Then, 0.35 g (1 mmol) [$(CH_3)_2$AuI]$_2$ was dissolved in 10 mL of cooled hexane and interacted with 0.22 g (1 mmol) of K(thd) obtained by neutralization of Hthd (97%, Dalchem, CAS 1118-71-4) by a KOH solution.

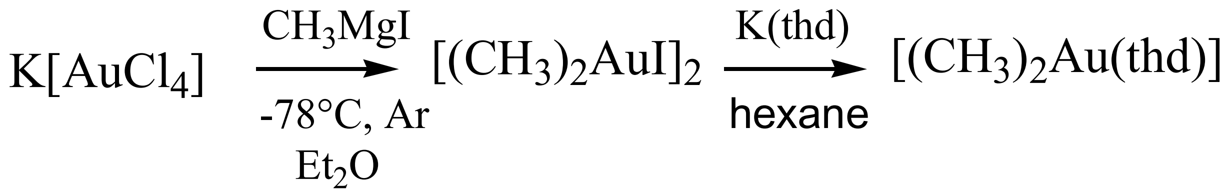


**Scheme S3.** Scheme of the synthesis of [$(CH_3)_2$Au(thd)].

The final product was purified by recrystallization from hexane; the yield of [$(CH_3)_2$Au(thd)] was 65% (0.26 g, 0.7 mmol). The elemental analysis data (CARLO-ERBA-11008, mass %): for $C_{13}H_{25}O_2Au$: found C 38.6; H 5.8; calc. C 38.1; H 6.1, melting point 74 °C.
